# Platelets enhance tissue factor protein and metastasis initiating cell markers, and act as chemoattractants increasing the migration of ovarian cancer cells

**DOI:** 10.1186/s12885-015-1304-z

**Published:** 2015-04-15

**Authors:** Renan Orellana, Sumie Kato, Rafaela Erices, María Loreto Bravo, Pamela Gonzalez, Bárbara Oliva, Sofía Cubillos, Andrés Valdivia, Carolina Ibañez, Jorge Brañes, María Isabel Barriga, Erasmo Bravo, Catalina Alonso, Eva Bustamente, Enrique Castellon, Patricia Hidalgo, Cesar Trigo, Olga Panes, Jaime Pereira, Diego Mezzano, Mauricio A Cuello, Gareth I Owen

**Affiliations:** 1 Departament of Physiology, Faculty of Biological Sciences, Pontificia Universidad Católica de Chile, Santiago, Chile; 2 Department of Obstetrics and Gynecology, Pontificia Universidad Católica de Chile, Santiago, Chile; 3 Division de Hematology & Oncology, Faculty of Medicine, Santiago, Chile; 4 Center UC Investigation in Oncology, Santiago, Chile; 5 Advanced Center for Chronic Diseases (ACCDiS), Pontificia Universidad Católica de Chile, Alameda 340, Santiago, Chile; 6 Hospital Sótero del Rio, Av. Concha y Toro 3459, Puente Alto, Santiago, Chile; 7 Hospital Gustavo Fricke, Viña de Mar, Santiago, Chile; 8 Fundación Arturo López Pérez, Av. Rancagua 878, Providencia, Santiago, Chile; 9 Institute of Biomedical Sciences (ICBM), Faculty of Medicine, Universidad de Chile Avda, Independencia 1027, Santiago, Chile; 10 Biomedical Research Consortium of Chile, Alameda 440, Piso 13, Santiago, Chile

**Keywords:** EMT, Coagulation, Metastasis initiating cells, Ascites, N-Cadherin, E-Cadherin, CD44

## Abstract

**Background:**

An increase in circulating platelets, or thrombocytosis, is recognized as an independent risk factor of bad prognosis and metastasis in patients with ovarian cancer; however the complex role of platelets in tumor progression has not been fully elucidated. Platelet activation has been associated with an epithelial to mesenchymal transition (EMT), while Tissue Factor (TF) protein expression by cancer cells has been shown to correlate with hypercoagulable state and metastasis. The aim of this work was to determine the effect of platelet-cancer cell interaction on TF and “Metastasis Initiating Cell (MIC)” marker levels and migration in ovarian cancer cell lines and cancer cells isolated from the ascetic fluid of ovarian cancer patients.

**Methods:**

With informed patient consent, ascitic fluid isolated ovarian cancer cells, cell lines and ovarian cancer spheres were co-cultivated with human platelets. TF, EMT and stem cell marker levels were determined by Western blotting, flow cytometry and RT-PCR. Cancer cell migration was determined by Boyden chambers and the scratch assay.

**Results:**

The co-culture of patient-derived ovarian cancer cells with platelets causes: 1) a phenotypic change in cancer cells, 2) chemoattraction and cancer cell migration, 3) induced MIC markers (EMT/stemness), 3) increased sphere formation and 4) increased TF protein levels and activity.

**Conclusions:**

We present the first evidence that platelets act as chemoattractants to cancer cells. Furthermore, platelets promote the formation of ovarian cancer spheres that express MIC markers and the metastatic protein TF. Our results suggest that platelet-cancer cell interaction plays a role in the formation of metastatic foci.

**Electronic supplementary material:**

The online version of this article (doi:10.1186/s12885-015-1304-z) contains supplementary material, which is available to authorized users.

## Background

Ovarian cancer is the deadliest gynecological cancer, causing around of 15,000 deaths in the USA this year [1]. Despite clinical advances, ovarian cancer continues to be a poorly understood disease with an unfavorable prognosis, principally due to late stage diagnosis when the cancer has already disseminated throughout the peritoneum [2]. The current theory of cancer progression proposes that from the total population of cancer cells within a primary tumor, only a small sub-population has the capacity to migrate, survive in isolation and establish secondary tumors within distant organs [3]. Metastasis Initiating Cells (MICs) are characterized by their enhanced chemoresistance, low metabolic rate, possessing “stemness”, for having undergone Epithelial-Mesenchymal Transition (EMT) and their enhanced capacity to generate metastatic foci. Cancer cell stemness (also known as cancer stem cells) refers to the presence of proteins on the cancer cells that are normally associated with physiological stem cells and whose presence associate with enhanced tumor-forming capacity in xenograft assays and increased chemoresistance [4] . These markers are numerous and differ depending on the tissue origin, however for ovarian cancer, one of the most reported examples is increased CD44 expression [5,6]. EMT, the conversion of epithelial cells to a more stromal or mesenchymal phenotype, is believed to be a fundamental event by which the cancer cell acquires migratory and invasive properties [7,8]. This event has been associated with lower E-Cadherin expression and enhanced N-Cadherin and vimentin levels [9]. The transcription factors Twist, Snail and Slug are known to promote EMT and cause the down regulation of E-Cadherin [10-12] As an *in vitro* model of MICs, under special culture conditions a heterogeneous population of cells can give rise to three-dimensional cancer spheres (cell clusters) which present enhanced expression of CD44 and display the capability of anchorage-independent growth [13].

Cancer patients have long been reported to present abnormal risk of thrombosis that correlates with the progression of the disease [14-16]. High platelet counts are prevalent in 31-42% of primary epithelial ovarian cancers and this correlated with significantly worse prognosis. [17-19].

It has been speculated that platelets may contribute to tumor metastasis through EMT induction [20], immune system evasion, adhesion to endothelial layer, and angiogenesis and vascular remodeling [21].

A further protein associated with cancer metastasis is Tissue factor (TF). This transmembrane receptor and initiator of the extrinsic coagulation pathway is not normally expressed in the vascular lumen, but makes contact with the circulatory system only upon vascular injury, resulting in clotting activation [22]. It is widely reported that many cancer types overexpress functional TF on the cell membranes and also in tumor derived microparticles, thus being responsible for enhanced coagulation and invasion [23-26].

Taken together, accumulating evidence is correlating platelet function with increased metastasis and poorer patient survival. However, to date, the effect of platelets on TF levels have not been described, neither are the levels of this protein during the acquisition of a MIC phenotype. Considering that ovarian cancer metastasis occurs mainly within the peritoneal cavity as a result of the accumulation of cancer cells in the ascites, the goal of the present work is to evaluate the effect of platelet interaction with ovarian cancer cells, regarding phenotype, TF and EMT associated protein levels and biological function. Herein, we present evidence that platelet addition brings about an increase in TF protein, a switch to a MIC phenotype and enhanced migration of ovarian cancer cells.

## Methods

### Human material

Ovarian ascites samples were obtained from the participating hospitals; Hospital Clínico Pontificia Universidad Católica de Chile (Santiago, Chile), Hospital Sótero del Río (Santiago Chile), Hospital Gustavo Fricke (Viña del Mar, Chile), Fundación Arturo López Pérez (Santiago, Chile). The cancer type and stage are included in Table 1. Human cancer cells were isolated from ovarian cancer ascites as previously reported [27-29]. Primary cultured cells in passage 2 were typically used for all experimentation. In the case of the benign ovarian fibrothecoma and the benign ovarian mucinous cystadenoma, cells were obtained from a peritoneal washing with physiological solution at 37°C prior to surgery. Platelets were obtained from healthy volunteers not taking medication that affects platelet function. All experiments and use of human samples were performed in accordance with the Declaration of Helsinki. Ethical committee approval was obtained from each participating hospital and regional health board. These include: the ethical committees of the Faculty of Medicine at the Pontifical Catholic University of Chile; Foundation Arturo Lopez Perez, Santiago Chile; the South Eastern Metropolitan Medical Service (SSMSO, Santiago de Chile); The Eastern Metropolitan Medical Service (SSMO, Santiago de Chile); the Quillota Medical Service (Region V, Chile). Informed written consent was obtained from all patients and blood donors.

**Table 1:** **Patient information**

Patient number	Stage	Classification	Surgery
**1**	IV	Ovarian serous papillary	Primary
2	II	Ovarian endometrioid adenocarcinoma	Primary
3	II	Ovarian serous papillary cystadenocarcinoma	Primary
4	IIIc	Primary peritoneal carcinomatosis	Primary
5	IIIc	Ovarian serous cystadenocarcinoma	Primary
6	IIIc	Ovarian endometrioid adenocarcinoma	Relapse
7	N/A	Ovarian benign fibrothecoma	Primary
8	IIIc	Ovarian mucinous cystadenocarcinoma	Primary
9	IIIc	Ovarian mucinous cystadenocarcinoma	Primary
10	N/A	Ovarian mucinous cystadenoma	Primary
11	IIIc	Ovarian serous papillary cystadenocarcinoma	Primary
12	IIIa	Ovarian mixed papillary serous and endometrioid mixed carcinoma	Relapse
13	IIIc	Ovarian adenocarcinoma (unspecified)	Primary
14	IIIc	Ovarian adenocarcinoma (unspecified)	Relapse
15	IIIc	Ovarian serous papillary	Primary
16	IV	Ovarian carninoma (unspecified, poorly defferentiated)	Primary
17	IIIc	Ovarian serous cystadenocarcinoma	Primary
18		Ovarian serous papillary	Primary

### Reagents

Insulin (SigmaAldrich, St. Louis, MO, Cat N° I2643-25MG), FGFb (Invitrogen Life Technologies, Carlsbad, CA, Cat N° AA 10–155, EGF (ProSpec, Ness Ziona, Israel, Cat N° CYT-217), PGE1 (SigmaAldrich, St. Louis, MO, Cat N° P5515). Human antibodies: CD44 (Santa Cruz Biotechnology, Santa Cruz, CA, Cat N° sc-7297), Tissue Factor (Calbiochem, San Diego, CA, TF9-10H10 (western-blotting), and American Diagnostica, Stamford, CT, Cat N° 4509 (flow cytometry)), N-Cadherin (Invitrogen Life Technololies, Carlsbad, CA, Cat N° 33–3900), E-Cadherin (Cell Signaling, Technology, NY, Cat N° 24E10), Histone 3 (Cell Signaling, Technology, NY, Cat N° 9715), Integrin β3 (Cell Signaling CD61, Technology, NY ,Cat N° 555753), IgG1 mouse (Cell Signaling, Technology, NY, Cat N° 555749), Vimentin (Millipore, Technology, NY, Cat N° MAB3400), Alexa fluor 555 (Invitrogen Life Technologies, Carlsbad, CA, Goat Anti-Rabbit IgG (H + L),Cat. N°: A-21428), Alexa fluor 488 (Invitrogen Life Technologies, Carlsbad, CA, Anti-Mouse IgG, IgM (H + L) Cat N° A-10680), HRP-conjugated anti-rabbit secondary antibody (Biorad, Goat Anti-Rabbit IgG (H + L)-HRP Conjugate; Cat. N°170-6515, USA) and β-Actin (Sigma Aldrich, St. Louis, MO, Cat N° A5060).

DNase I (Invitrogen Life Technologies, Carlsbad, CA), RNAse H, dNTP mix, EDTA, DTT, Tris–HCl KCl, MgCl_2_ were all purchased from Invitrogen Life Technologies, Carlsbad, CA. Fast SYBR green master mix was bought from Applied Biosystems, USA (Cat no. 4385612). Nuclease free water was purchased from Winkler, Santiago, Chile. Factor VIIa, Factor X, chromogenic substrate “Spectrozyme FXa”, TFPI-1 (222B, 526, 526 and 4900 American Diagnostica, Stamford, CT, respectively), Low Attachment plates (Corning, NY Cat N° 3471), Fluoromount (Electronic Microscopy Science, Hatfield, PA, Cat N° 17984–25) and Boyden chambers (Corning, Cat N° 3458), phototgraphic film (Thermo Scientific™ CL-XPosure™ Film; Cat. N° PI-34091), Ponceau-S red stain (Sigma Aldrich, St. Louis, MO), Hoechst (Invitrogen Life Technologies, Carlsbad, CA, Cat N° H1399) and Fluoromount (EMS 17984–25). Primers, listed in Table 2, were purchased from IDT, USA.

**Table 2:** **Sequence of the primers used for mRNA levels analysis**

Sequence 3′ > 5′	GENE
	**N CADHERIN**
Forward primer	ATTTCCATCCTGCGCGTGAA
Reverse primer	TTGTTTGGCCTGGCGTTCTT
	**E CADHERIN**
Forward primer	TTTCTTCGGAGGAGAGCGGT
Reverse primer	ATGAGGGTTGGTGCAACGTC
	**CD44**
Forward primer	CCCAGACGAAGACAGTCCC
Reverse primer	GCCTCTTGGTTGCTGTCTCA
	**TISSUE FACTOR**
Forward primer	CCAAACCCGTCAATCAAGTC
Reverse primer	ACAATCTCGTCGGTGAGGT
	**HPRT1**
Forward primer	GACCAGTCAACAGGGGACAT
Reverse primer	ACACTTCGTGGGGTCCTTTTC

### Extraction of human platelets

Venous blood (64 mL) was collected from healthy volunteers (not taking anti-platelet drugs) in ACD-A (1:10, vol/vol). After centrifugation (9 minutes at 200 g), the top two-thirds of platelet-rich plasma (PRP) was removed and re-centrifuged (9 minutes at 2700 g). The pellet was washed in buffer (137 mM NaCl; 2.68 mM KCl; 11.9 mM NaHCO3; 0.36 mM Na_2_HPO_4_x2H_2_O; 2 mM MgCl_2_, [pH 6.2]) containing prostaglandin E1 (PGE1) (120 nM), then centrifuged twice (200 g for 9 minutes) to remove residual leukocytes. Leukocyte contamination was evaluated by fluorescence microscopy using propidium iodide as a nuclear stain. Leukocytes counts were always less than 1x10^5^ platelets. All centrifugation steps were performed at 4°C. Platelet concentration was determined using the Coulter Particle Counter WS-Z1DUALPC. Co-culture of human washed platelets and ovarian cancer cells. Human platelets were centrifuged (9 minutes at 2700 g) at 4°C, supernatant was discarded and the pellet was suspended in 3 mL of DMEM F12 5% charcoal treated serum medium at 37°C. Platelet-containing medium was added to the culture dish (final concentration 150,000 platelets/μL) containing already seeded cell lines A2780, UCI101, SKOV3 and the human ascites primary culture. After 6 or 12 hours of incubation, the cell monolayer was washed three times with PBS to eliminate the supernatant platelets.

### Ovarian cancer sphere formation assay

200,000 ovarian cancer cells (UCI101), were cultured in 6 well low attachment plates in DMEM/F12 medium supplemented with FGF 50 μg/mL; EGF 0.2 mg/mL; insulin 5 g/mL. Every 48 hours 1 mL of supplemented medium with or without 150,000 platelets/μL was added. After 7 day of culture, medium was passed through a 100 μm strainer to collect ovarian cancer sphere clusters (the size of spheres typically ranges 40–100 μm in diameter) [30]. To be sure that we excluded aggregates, spheres were recuperated, counted and analyzed by flow cytometry. Spheres were counted by microscopy at 20X, using 17 fields with covered the entire plate.

### Scratch and migration assay

UCI101 and SKOV3 cells were cultured in standard tissue culture plates until 80% confluence, and then a vertical wound (scratch) was introduced through the cell monolayer using a fine pipette tip. The culture medium was replaced with fresh DMEM/F12 containing 5% charcoal treated serum in the presence or absence of platelets (150,000 platelets/μL). Wound closure was assessed by photography at 12, 24 and 48 hours and quantified using the Image J software.

Chemoattractant effect was further analyzed using Boyden chambers. To these chambers, 7.5 x 10^4^cells were seeded in the upper well in 400 μL of DMEM/F12 5% charcoal treated serum medium, while the lower well was filled with the same medium. Platelets were added in the upper or the lower well (150,000 platelets/μL) as stated in the figure legends. Corresponding controls without platelets were performed. After 24 hours of incubation at 37°C and 5% CO_2_ the membrane of the upper chamber was cut and fixed with methanol at −20°C during 20 min. The membranes were washed 3 times with PBS, and then the endogenous peroxidase was inhibited by using hydrogen peroxide for 10 min at darkness. Then membranes were blocked for 2 hours with PBS-BSA 1% and incubated with vimentin antibody (1:100) over night. Membranes were washed 3 times with PBS and revealed with immunocytochemistry kit (DAKO) according to the procedures manual. Finally we counted the number of cells that migrated through the membrane using a light microscope.

### Flow cytometry analysis

With full ethical committee approval, during surgery the ascites is extracted through a small puncture made in an avascular zone of the peritoneum to minimize contamination with blood. Once the liquid is extracted from the cavity, the rest of the abdomen is opened for surgery. Cases of hemorrhagic ascites were not used in this study. For analysis of human ovarian ascites samples (all samples came from cancer patients post-menopause), 1 mL of fluid was collected and centrifuged during 3 min at 3000 rpm. Then the supernatant was centrifuged for 9 min at 2700 g and the pellet was resuspended in 300 μL of 1% BSA and separated into 3 aliquots. The first was used as a negative control. The second tube was incubated with 5 μL of Anti CD61 and 5 μL of normal mouse IgG; and the third tube was incubated with 5 uL of Anti CD61 and 5 μL of Anti CD62P. After 25 min in darkness and room temperature the tubes were thrice washed in PBS. Samples were read in the flow cytometer (ACCURI C6), and the data analyzed using the software C Flow Sampler. For the co-culture of platelets and cancer cells, the platelets were added over the cells for 15 min. Later 1 mL of medium was centrifuged for 9 min at 2700 g and the pellet suspended in PBS-BSA 1%. Then the platelets were analyzed for CD61 and CD62P markers. For TF analysis 5 μL of TF antibody was added for 25 min at room temperature, followed by 1 μL (from a previous 1/10 dilution) of Alexa 647 anti-mouse for 20 min in darkness. Cells were subsequently washed for flow cytometer analysis.

### Western blot analysis

120,000 cells were seeded in 6 cm plates. Total proteins from cell lines were extracted using 150 mM Tris–HCl lysis buffer containing NaCl 1.5 M, and Triton X-100 0.5%. Lysed cells were left for 20 minutes on ice, centrifuged at 4,000 rpm for 5 minutes at 4°C, then cells are sonicated and centrifuged at 4,000 rpm for 5 minutes at 4°C. The resulting protein content of the supernatant was determined by the Bradford method and stored at −80°C. Equal amounts of protein (100 μg/lane) were separated using 12% SDS-PAGE or 10% SDS-PAGE under reducing conditions and transferred to nitrocellulose membranes (Biorad, Hercules, CA), blocked with BSA 3% in TBS-0.1% Tween-20 and incubated overnight at 4°C with the primary antibodies anti- E-Cad (1:500), anti-N-Cad (1:500), and anti-CD44 (1:500). For TF analysis we blocked with 5% nonfat dry milk in TBS-0.1% Tween-20 buffer (TBS-T) and incubated overnight at 4°C with anti- TF (1:500). For β-actin (1:3000) analysis and Histone H3 (1:1000) we blocked with 5% nonfat dry milk in PBS-0.1% Tween-20 buffer (PBS-T) both used as load control. All the antibodies were diluted in blocking buffer. The membranes were washed three times for five minutes in TBS-T buffer, incubated with HRP-conjugated anti-rabbit secondary antibody for two hours at room temperature and developed with chemiluminescence reagent (Pierce ECL Thermo Scientific, US.). Membranes were exposed to phototgraphic film, with equal protein loading confirmed with Ponceau-S red staining. Images were scanned at 16-bit/600 dpi resolution with an Epson Perfection 3490 scanner (Epson Corporation, CA), saved as TIFF files and calibrated to an optical density scale. The integrated optical density of bands was quantitated using the Image J v.1.47 software. The optical densities were expressed as the ratio of treatment/control.

### Immunofluorescence

From ascites extracted with informed consent from ovarian cancer patients, 1 mL was placed onto a coverslip and allowed to evaporate at room temperature. The slide was fixed with methanol at −20°C during 20 min before blocking with 2% BSA for 30 mins. Antibodies against CD42b (reacts with GpIb specific for platelets, 1:250, and vimentin (cancer cells, 1:1000,) were applied overnight in the dark at 4°C. The slide was washed 3 times with PBS and the secondary antibody (anti-mouse Alexa 488) or anti- rabbit (Alexa Fluor 555, Goat Anti-Rabbit IgG (H + L)) were used at a dilution of 1:10000 for 1 hour in the dark at room temperature. Cells were washed 3 times with PBS and then stained with Hoechst (1:50000) for 4 minutes. The slides were mounted in Fluoromount and observed under a NIKON Eclipse E200 fluorescent microscope.

### Pro-coagulant activity assay

After 24 hours of co-culture cells were washed with PBS, detached using trypsin and washed three times again to remove remaining platelets. Then, 5 x 10^4^ cells were placed into 96 wells plate. The cells were incubated in presence of FVIIa (1U mL^−1^), FX (1,2 U mL^−1^), CaCl_2_ (25 mM) and Spectrozyme FXa (1 mM) for 30 min. TFPI-1 (0.5 ug/ml) was added 30 minutes prior to the coagulation factors. Optical density was read in microplate spectrophotometer (EPOCH, Model 251450, BioTek, USA) at 405 nm.

### RNA extraction

150,000 ovarian cancer cells were seeded for these experiments. Total RNA was extracted from co-cultures of ovarian cancer cell lines and primary cultures of ascites using Trizol (Invitrogen Life Technologies, Carlsbad, CA). Concentration and purity was evaluated through optic density at 260 nm and the 260 nm/280 nm ratio (EPOCH, Model 251450, BioTek, USA). RNA was suspended in nuclease free water and its integrity confirmed by the presence of intact 18 s and 28 s ribosomal subunits by agarose gel electrophoresis.

### RT-PCR

2 μg of total RNA was treated with DNase I (1U μL^−1^) for 10 min at 25°C to eliminate possible contamination with genomic DNA. The reaction was stopped with 1μL of EDTA (25 mM, pH 8) at 65°C for 15 min. The reverse transcriptase reaction was performed using a mix composed of 2 μL of random primers (50 ng μL^−1^, Invitrogen Life Technologies, USA) was added to 1 μL of dNTP mix (ATP, CTP, GTP, TTP, 10 mM) , and incubated at 65°C for 5 min. Subsequently, 4 μL of reverse transcriptase buffer 5 X (250 mM Tris–HCl pH 8.3; 375 mM KCl, 15 mM MgCl_2_), 2 μL of 0.1 M DTT and 1 μL of Superscript reverse transcriptase II and RT-RNAse H- (200 U μL^-1^) were added and incubated at 25°C for 10 min, followed by 50 min at 42°C for, and finally 15 min at 70°C in an Axygen Maxygene thermocyler.

### Real time PCR

To evaluate mRNA levels in cell lines and primary cultures, 2 μg of cDNA was incubated with: 10 μL of Fast SYBR green master mix (Cat no. 4385612, Applied Biosystems, USA), 7 μL of nuclease free water, 1 μL of primers (250 nM, IDT, USA). All of the amplifications obtained had a unique peak for each PCR product. The sequence of each primer set used is shown below in Table 2. To perform this analysis we used the 7500 fast real-time PCR system (Applied Biosystems, USA). For the analysis the program consisted of an initial step of 95°C for 20 sec, then 40 cycles of 3 sec at 95°C and an annealing/extension step of 30 sec at 60°C in which the reporter signal was acquired. The melting curve was formed by: 95°C for 15 sec, followed by 1 min at 60°C, then 30 sec at 95°C and finally 15 sec at 60°C. The values obtained were normalized relative to HPRT1 levels and then the results were graphed using the method of 2^-( ΔCt)^, data is represented as the log of the mean relative to control.

### Statistical analysis

The statistical analysis was performed using GraphPad Prism 5 software (GraphPad, Inc., San Diego, CA). The results are expressed as means ± standard deviations. The differences in the results showed in the scratch and chemoattractant assays were analyzed using ANOVA test with Bonferroni Post-test. The differences in the results showed in the levels of MICs markers and TF, and in number of spheres were analyzed using the student’s t test. P values of <0.05 were considered statistically significant.

## Results

**Figure 1:**
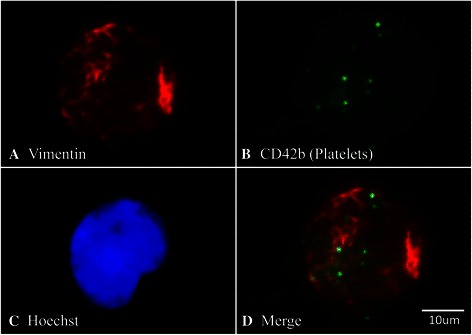
Platelets are present in ascites from ovarian cancer patients. Representative immunofluorescence from a smear of ascites obtained from ovarian cancer patients. Platelet presence is shown by the platelet specific antibody CD42b (green, panel **B**) and merged with vimentin antibody **(PanelA)**, Hoechst stain **(Panel C)** and merged image **(panel D)**. Image (40X).

Immunofluorescence using a platelet specific CD42b antibody (that reacts with platelet GPIb) demonstrated that platelets are present together with cancer cells in peritoneal fluid extracted from an ovarian cancer patient (Figure 1). The merge with vimentin antibody and Hoechst stain shows that the platelets associate with cancer cells (see also immunofluorescence negative control in Additional file 1: Figure S1). Furthermore, flow cytometer analysis of ascites extracted from individual patients demonstrated the presence of free platelets in this fluid (see Additional file 2: Figure S2).

**Figure 2:**
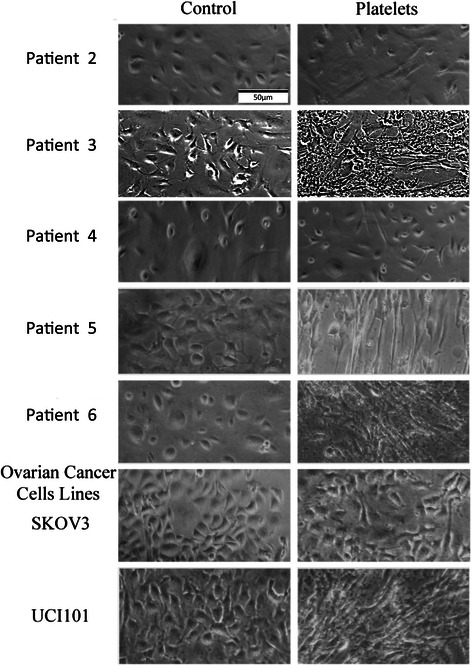
Platelets modify ovarian cancer cell phenotype. Light microscope images (20X) of ascites primary cultures and ovarian cancer cell lines co- cultured with or without platelets (150,000/μL) during 12 hours.

All patient information is confidential and thus coded for scientific use. We assigned patient numbers (e.g. patient 5) to cancer cell populations isolated from individual patients to allow comparison of results between the different assays used in the paper. Due to limited cell numbers, not all experiments could be performed for each patient. After confirming that platelets are present together with cancer cells, we moved to an *in vitro* model to determine the effect of platelets on primary cultures of advanced ovarian cancer cells. The co-culture of ascites from 5 separate advance ovarian cancer patients for 12 hours with 150,000 platelets/uL (physiological concentration) resulted in a marked change in cancer cell phenotype, with the occurrence of cell elongation and a more stromal appearance (Figure 2). It was noted that the majority of platelets become activated upon contact with cell culture medium (please refer to Additional file 3: Figure S3). The change in phenotype was present in each patient culture tested, however different degrees of phenotypic change were noted (compare patient 2 and 4 with patient cultures 3, 5 and 6) (Figure 2). This was also reflected in ovarian cancer cell lines treated in the same manner, where SKOV3 had subtle changes, whereas UCI101 showed a rapid and notorious phenotypic alteration.

**Figure 3:**
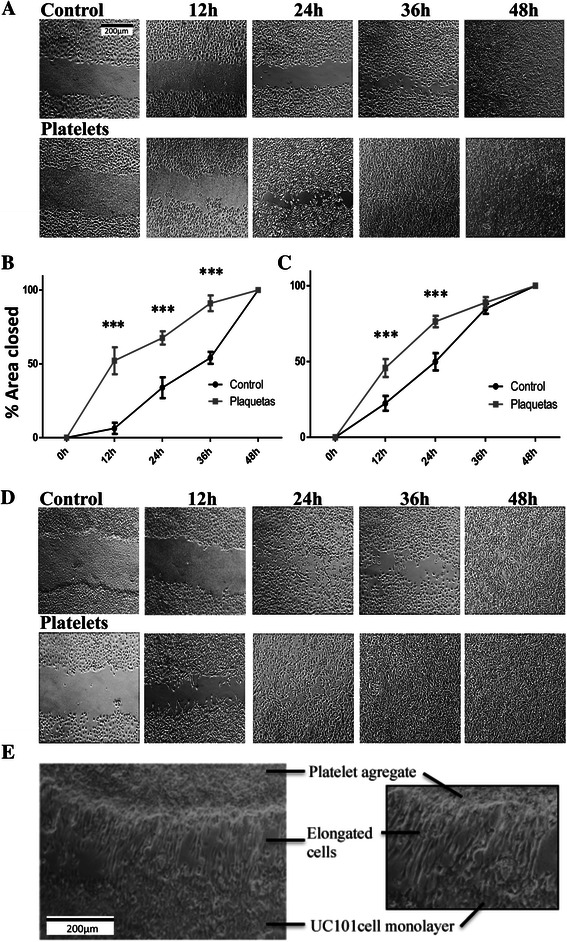
Platelet enhance ovarian cancer cell migration. Scratch assay of ovarian cancer cell lines treated with or without platelets (150,000/μL). **A** and **C**: SKOV 3 and UCI101 images (10X) of scratches at 0, 12, 24, 36 or 48 hours. **B** and **D** quantification of the percentage of area respect to the initial scratch area (Image J) N = 3; ***p < 0.001 respect to control, ANOVA with Bonferroni as Post- test. **E**: Light microscope image (10x) of the interaction between platelets and UCI cells after 12 hours of co-culture with platelets.

**Figure 4:**
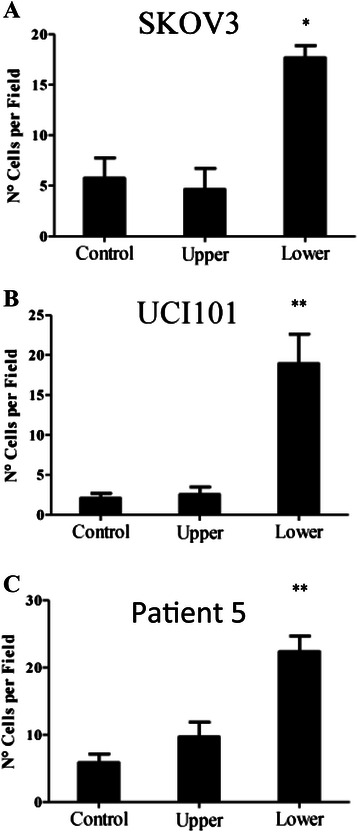
Platelets act as a chemoattractant to enhance ovarian cancer cell migration. Effect of platelets on migration and chemotaxis assays performed in SKOV3, UCI101 and primary cultured cancer cells obtained from ovarian ascites (Patient 10). **A**, **B** and **C** shows the number of cells that migrated under “Control” (no platelets added), “Upper” (platelets added within the cells) or “Lower” conditions (platelets added in the lower chamber) N = 3 for cell lines, **p < 0.01 relative to control, ANOVA with Bonferroni as Post- test.

Given that the fibroblastic phenotype has been correlated with migratory potential, we tested if platelet presence enhanced cell motility. The scratch assay disclosed an enhanced ovarian cancer cell migration at 12 and 24 hours post-addition of platelets (Figure 3). Cell cycle analysis by flow cytometry demonstrated that the platelet-mediated increase in migration was not a result of increased cancer cell proliferation (please refer to Additional file 4: Figure S4). Interestingly, during the course of these migration assays we visually observed that the cancer cells (especially in the UCI101 cell line) appeared to be attracted to platelet aggregates that had formed in the culture plate. This is shown at different magnifications (Figure 3E), where the ovarian cancer cells nearest to the platelet aggregate are more elongated. To test the hypothesis that platelets have chemoattractant capacity, we performed migration assays using Boyden chambers. In this experiment ovarian cancer cells SKOV3 (Figure 4A), UCI101 (Figure 4B) or primary cultured ascites cells (Patient 5, Figure 4C), were placed with platelets in either the upper chamber or in the lower chamber below the polycarbonate membrane and not in contact with the cancer cells. As shown in each of the three experiments, an increase in migration to the lower side of the polycarbonate membrane was only observed when the platelets were used as a chemoattractant, independent of platelet-induced phenotype changes (Figure 2).

**Figure 5:**
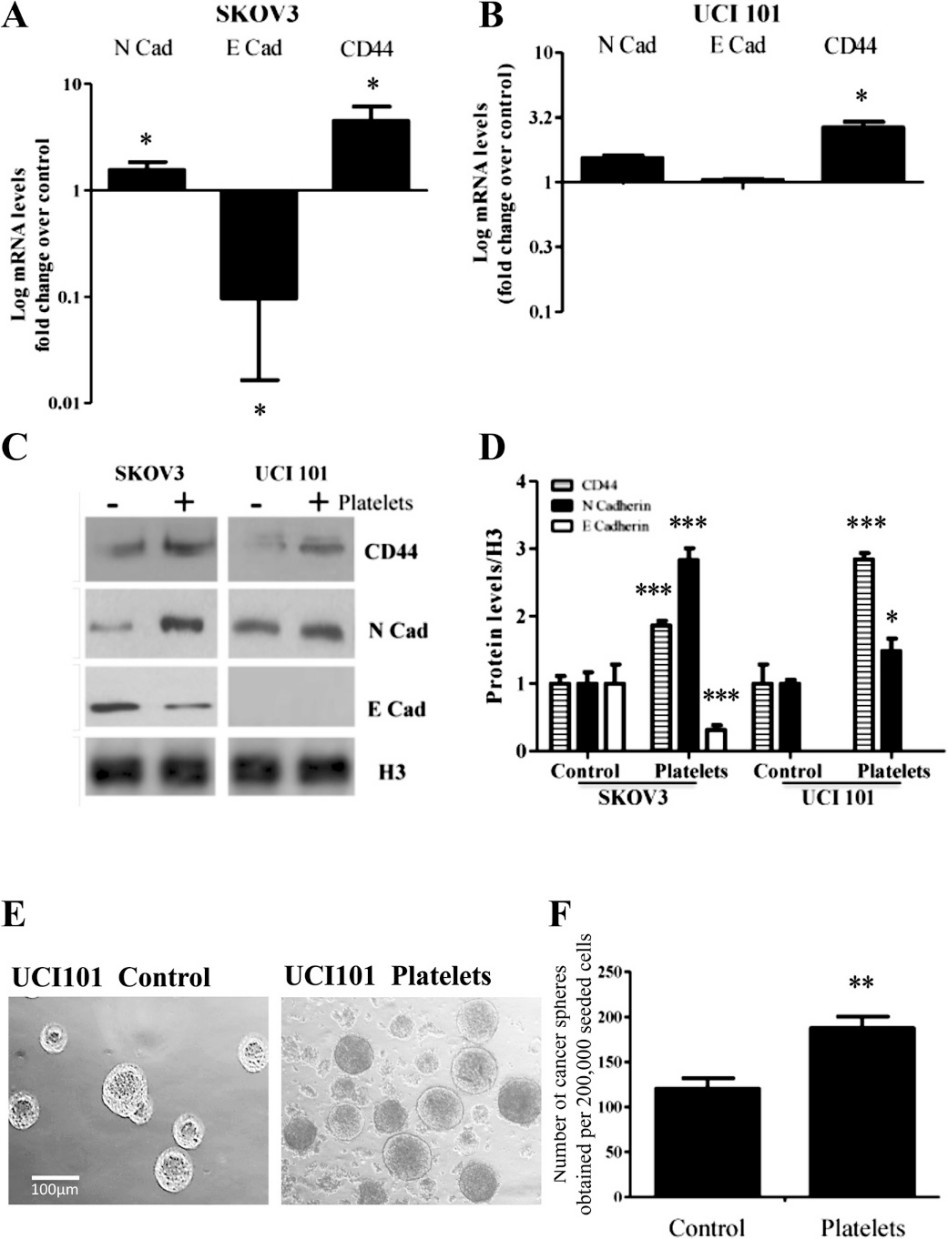
Platelets increase MICs marker levels and ovarian cancer sphere formation. mRNA levels in respect to control of SKOV3 **(Panel A)** and UCI101 **(Panel B)** cell co-cultured during 6 hours with platelets (150,000/μL, N = 3). Data is represented as the log of the mean relative to control. Representative images of protein levels of ovarian cancer cell lines after 12 hours of co-culture with platelets **(Panel C)** with quantification in **panel D** (N = 3) representative images of ovarian cancer spheroids (spheres) formed by UCI101 cells in the presence and absence of platelets **(Panel E)**, with quantification shown in **Panel F** (N = 3), right: quantification of the total number of spheres counted *p < 0.05 ; **p < 0.01; ***p < 0.001, student t-test.

To analyze further the phenotype change we examined at the transcript and protein level genes known to be associated with a MIC and EMT phenotypes. In both cell lines tested, platelet co-culture for 6 hours increased CD44 and N-Cadherin mRNA levels. E-Cadherin was barely detectable in UCI101, but was decreased in SKOV3 (Figure 5A and B). This mRNA change was also reflected at the protein level, as demonstrated by representative blots (Figure 5C), and in its quantification (Figure 5D). A platelet-induced increase in the EMT markers Snail and Twist was also observed in SKOV3 cells and primary cultured cancer cells (please refer to Additional file 5: Figure S5).

Culture of the ovarian cancer cell line UCI101 in basal medium was used to form cancer spheres in the presence and absence of platelets. Isolated spheres were selected on the basis of size and were confirmed to demonstrate increased expression of CD44 by immunocytochemistry and expression of EMT protein markers by Western blotting (please refer to Additional file 5: Figure S5). Spheres formed under both conditions; however their number was greatly enhanced in the presence of platelets (Figure 5E and F).

**Figure 6:**
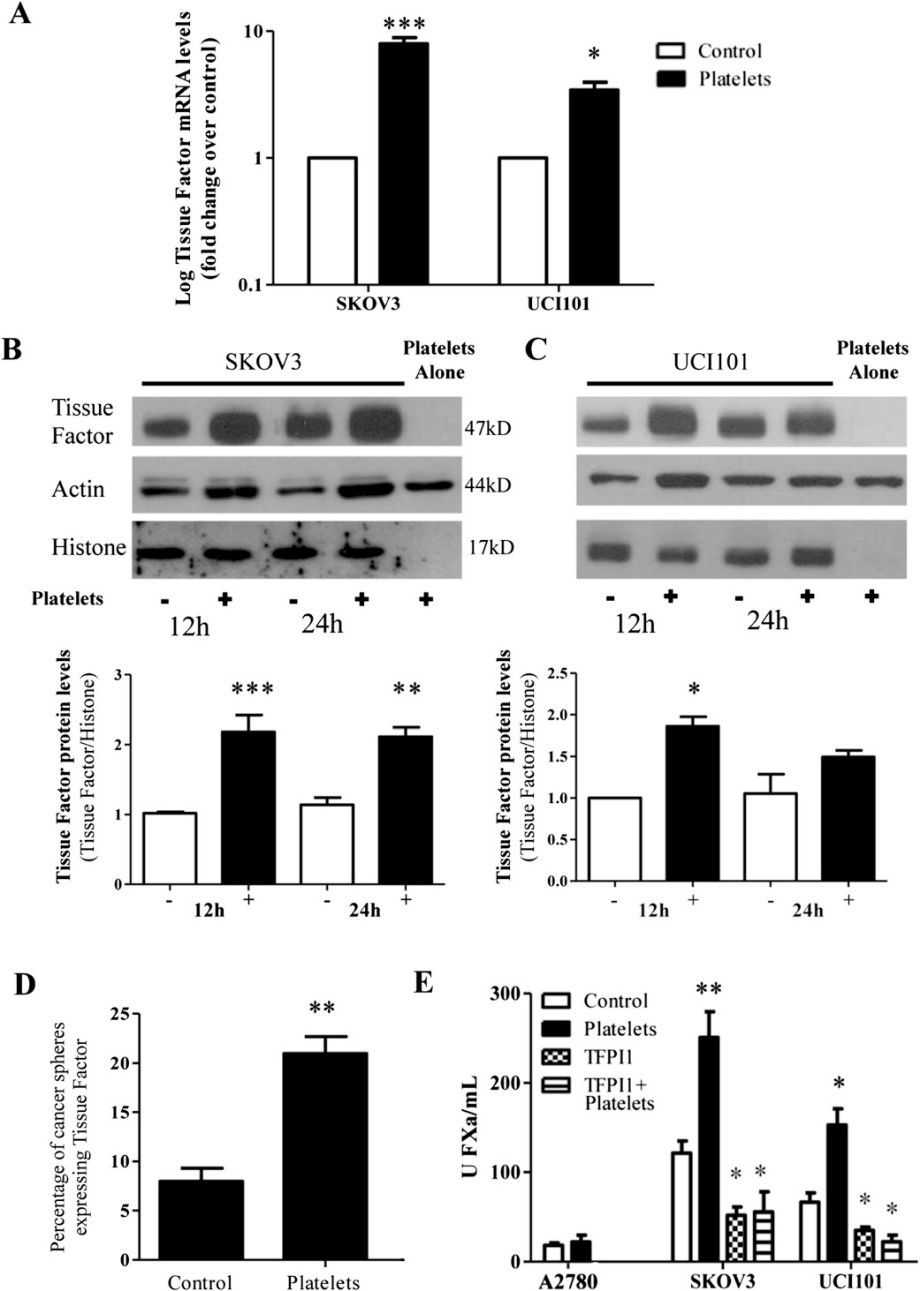
Platelets increase Tissue Factor (TF) levels. **A**: Levels of mRNA relative to control of ovarian cancer cell lines SKOV3 and UCI101 co-cultured with platelets for 6 hours (N = 3). **B** and **C**: Western blot analysis of cell lines SKOV3 and UCI101 co-cultured with platelets during 12 or 24 hours. Above: density analysis of western blot bands respect to H3 levels. (N = 3). **D**: Flow cytometry analysis of TF levels of ovarian cancer spheroid of UCI101 cells formed in presence or absence of platelets (N = 3) **E**: Procoagulant activity assay (units of FXa formed) of ovarian cancer cell lines cultured during 24 h with or without platelets (N = 3). *p < 0.05; **p < 0.01; ***p < 0.001 respect to control, student´s t-test.

Platelet interaction with tumor cells increased TF mRNA at 6 hours (Figure 6A) and the corresponding protein levels at 12 and 24 hours (Figure 6B and C, for SKOV3 and UCI101, respectively). β-Actin expression in these conditions is also increased due to the presence of platelets (Figure 6B and C). To control for this we evaluated the nuclear protein Histone 3 which is not expressed by platelets (Figure 6B and C) as a loading control for the levels of cancer cell proteins. This increase in TF protein upon platelet interaction was also observed in cells isolated form spheres (Figure 6D). By flow cytometry we observed that in the whole cell UCI101 population 40% of the cells expressed TF. Of the population of UCI101 cells that formed spheres, only 8% expressed this protein, however this percentage increased to 22% in sphere cells that formed in the presence of platelets. The increase of TF protein in tumor cells was associated with an enhanced TF-mediated FXa generation, both in SKOV3 and UCI101 cell lines (Figure 6E). Despite platelets having been reported to express functional TF [31] our antibody did not detect TF protein in the platelet lysates (Figure 6B and C). Analyzing the ovarian cancer line A2780 that does not express TF, we observed no changes in procoagulant activity (Figure 6E) after incubation with platelets. The tissue factor pathway inhibitor (TFPI-1) inhibited the increase in coagulation in SKOV3 and UCI101 cells demonstrating that TF was responsible for the platelet-mediated increase in coagulation (Figure 6E).

**Figure 7:**
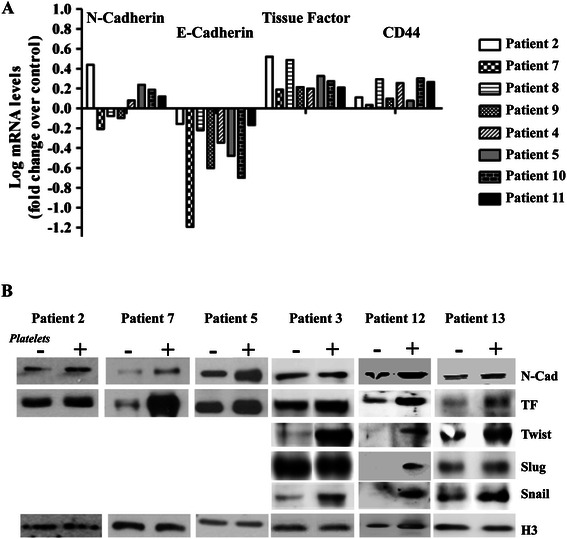
Platelets modify MIC marker and TF levels of primary cultured cancer cells extracted from the ascites of ovarian cancer patients. **A**: mRNA levels of MICs markers and TF over control of 8 patient samples cultured with or without platelets for 6 hours. Data is represented as the log of the mean in respect to control. **B**: Western blot analysis of N-Cadherin, TF, Twist, Slug and Snail in primary cultures treated with or without platelets during 12 hours. Protein yields were not sufficient to analyze all proteins in the primary cultures.

**Figure 8:**
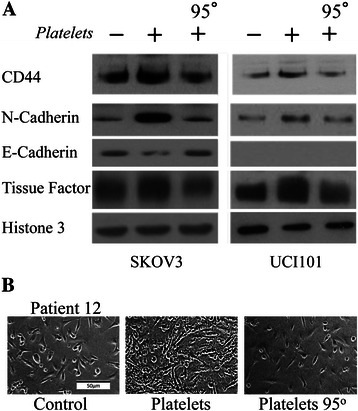
Denatured platelets do not alter protein levels or bring about a phenotypic change in ovarian cancer cells. **A**. Representative Western blots of SKOV3 and UCI101 cells co-cultured during 24 hours with either vehicle, platelets (150,000/μL) or platelets heated to 95°C for 5 minutes prior to addition. **B**. Representative light microscope images (20X) of primary cultured cancer cells (patient 13) co-cultured for 12 hours with either vehicle, platelets (150,000/μL) or platelets heated to 95°C for 5 minutes prior to addition to culture medium.

To demonstrate that the changes in TF and MIC/EMT markers with platelets were not a cell line artifact, we obtained primary cultures of ovarian cancer cells from eight separate patients. After 6 hours of platelet addition to these cancer cells we observed an increase in TF and CD44, with a decrease in E-Cadherin in all patients. An increase in N-Cadherin was observed in 5 of the 8 patients (Figure 7A). Although in many primary cultures not enough cells were recovered for western blotting, we demonstrate that these changes at the level of mRNA were also reflected at the protein level (Figure 7B). Interestingly, primary culture of a benign ovarian fibrothecoma (Patient 7, Figure 7A), a benign ovarian mucinous cystadenoma (Patient 10, Figure 7A) and a primary peritoneal carcinomatosis (Patient 4, Figure 7A), demonstrated that platelet-induced regulation of E-Cadherin, TF and CD44 was not limited to only malignant ovarian cancers. Furthermore, in three of three patients analyzed we observed a platelet-mediated increase in the EMT-related proteins Twist and Snail (Figure 7B). Finally, to evaluate if the effects observed by platelets are exclusively due to the presence of platelet membrane or whether factors released from the platelets (or membrane associated factors) are required, we denatured the platelets by heating to 95°C before addition to the culture medium. As observed in Figure 8A, denatured platelets are incapable elevating CD44 and N-Cadherin, or decreasing E-Cadherin protein levels in ovarian cancer cells. The platelet-induced change in phenotype of ovarian cancer cells was also lost (Figure 8B).

## Discussion

Metastasis is not solely a function of the tumor cells. The cancer cell requires an intricate coordination with its immediate environment, the site of extravasation and the host tissue (reviewed in [32]). The observation that cancer cells come into contact with platelets has been already established. Confocal microscopy analysis of leiomyosarcoma and histiocytoma tumors has demonstrated the presence of platelets and activated platelets within the tumor mass [33]. In a mouse cancer model, platelets expressing yellow fluorescent protein (YFP) isolated from female transgenic C57BL/6 mice were transfused by tail-vein injections into ovarian tumor-bearing mice. The presence of extravascular YFP platelets was observed in both ascites and tumor specimens [17]. Furthermore, once the cancer cell leaves the solid tumor and enters the blood stream platelet-cancer cell interaction becomes inevitable. Preclinical data suggest that cancer cell-platelet interaction in the blood facilitates tumor metastasis. These platelets may form aggregates with circulating tumor cells and physically act as a shield for the host immune surveillance [34]. A report by Stone and colleagues (2012) demonstrated a median time to disease progression of 4.65 years in patients without thrombocytosis, as opposed to 2.62 years in patients presenting more than 450,000 platelets/uL [17].

The platelet concentration present in ascites is difficult to ascertain, as platelets entering the peritoneal cavity would be expected to adhere to collagen in the peritoneal wall. In fact, in mouse models it has been shown that circulation-derived platelets adhere to the peritoneum surface [17]. We also see that a high proportion of the platelets that enter the ascites are bound to cancer cells (Figure 1). For these reasons we utilized normal circulating concentrations of platelets in our studies. Furthermore, this concentration of platelets allows us to extrapolate our proposed model to cancer cells entering the blood stream.

It has been convincingly demonstrated that the platelet addition to tumor cells can induce an invasive mesenchymal-like phenotype and that platelets can prime the tumor cells for metastasis [20]. Herein, we take this story a step further, demonstrating that this also occurs in ovarian cancer cell lines and in primary cultured cancer cells extracted directly from patients. In accordance with previous reports in the literature, our platelets underwent activation upon contact with the cancer cell cultures and we also detected high levels of platelet activation in patient ascites (Additional file 2: Figure S2 and Additional file 3: Figure S3) [35]. In all patient-derived cancer cells (8 patients) we found a reduction in E-Cadherin and an increase in the stem cell marker CD44 and TF protein upon exposure to platelets. This loss in E-Cadherin may be due to the platelet-mediated increase in Twist and Snail observed in Figure 7 [10-12]. We are not aware of previous reports describing increases of CD44 and TF after platelet exposure in ovarian cancer. We have previously demonstrated in breast cancer cells that increased TF expression is associated with enhanced cancer cell migration [25]. CD44 and a stemness phenotype have also been reported by others to increase migration and invasion [36-40]. Accordingly, in this manuscript we demonstrated statistical differences in migration of ovarian cancer cells upon exposure to platelets (Figure 3). An increase in migration by platelets has been observed previously in pancreatic cancer cell lines and this effect was reduced by the antiplatelet cilostazol [41]. However, a strikingly novel observation of our study was the attraction of ovarian cancer cells to platelet aggregates. In Boyden chambers we showed that the presence of platelets below the tumor cells caused enhanced migration across the polycarbonate membrane, thus a physical movement of tumor cells towards the platelets. This suggests that along with a change in EMT markers, the observed change in phenotype may be a result of cells migrating towards platelets.

Interestingly, the ability of platelets to mediate cancer cell proliferation has been demonstrated in the literature, however the majority of our observations were performed at time points below the doubling time of these cancer cell lines [42]. We demonstrate at the 12 hours time point used in the migration assay that there was no change in cell cycle upon platelet addition (Additional file 4: Figure S4) and thus we speculate that platelets increase the migration of ovarian cancer cells through chemoattraction. Whether the expression or EMT and/or TF are involved in this chemoattraction is still unknown. Interestingly, the presence of the platelets above the cancer cells did not increase a downward migration, despite an increase in lateral migration being observed in the scratch assay. This may add evidence to our theory that the movement is towards the platelets (chemoattraction). In the scratch assay we did notice enhanced migration when a platelet aggregate was in the scratch area. The possibility that platelets can be chemoattractant to cancer cells *in vivo* raises several questions. Could platelet accumulation at sites of vascular injury attract or capture cancer cells in circulation? Once at this site, could the enhancement of EMT markers and TF promote extravasation? These questions may be also valid in the opposite direction; platelets at areas of vascular injury or poor vascular integrity (as often observed in tumors), could attract cancer cells to escape from the primary tumor and migrate towards the vasculature and thus become metastatic. These scenarios seem attractive in the setting of ovarian cancer where it is known that ascites derived platelets adhere to the peritoneal membrane, where the metastatic foci of ovarian cancer occurs.

Platelets contain many molecules with attractant properties, such as PDGF [43] or TGFβ [44], among others, that may play a role in this effect. Platelets have also been shown to liberate microparticles that could be playing a direct role upon the cancer cells [45]. Although we demonstrate that denatured platelets cannot bring about a change in phenotype and EMT marker protein expression, further experiments are required to elucidate the pathophysiological contribution of platelet-induced attraction in the development of metastasis. However, platelet-derived TGFβ1, together with other platelet-bound factors have been shown to cooperate to increase metastasis. Platelets can activate the NF-κB signaling pathway in a contact-dependent manner. Interestingly, it appears that platelet-induced effects on EMT and invasion are not mediated by secreted TGFβ alone, but require additional platelet-bound factors [20].

An approach to identify MICs *in vitro* is through the use of sphere-forming assays. Under special culture conditions, a subset of a cancer cell line or primary tumor population cells are able to form three-dimensional spheres, demonstrate enrichment in stem cell markers and display the capability of anchorage-independent growth. CD44-positive spheres isolated from ovarian serous adenocarcinomas by this technique have been shown to form tumors more efficiently in animal models [13]. Using this method we noted that the presence of platelets enhanced the number of spheres formed. In the whole cell population of the UCI101 ovarian cancer cell line, 40 % of these cells expressed TF. Only 8% for the spheres that formed expressed TF, however in the presence of platelets the fraction of TF-expressing spheres increased to 22%. The representation of a “metastasis initiating cell” with a rigid definition of protein markers is a concept that is mostly likely going to change. Of the numerous MICs that leave the primary tumor, most probably only a few of these are capable of forming secondary tumors. It is also likely that the MICs that survive to form metastatic foci change the expression of their cell surface proteins numerous times before the establishment of metastatic foci. For example, the presence of TF may or may not be necessary for the MIC to leave the primary tumor, but it may be necessary to cause localized coagulation to recruit more platelets to shield the cancer cell from the immune system [46]. Strengthening this theory, it has been reported that pre-operative serum TF levels of ovarian cancer patients are around 85.2 pg/mL, significantly higher than levels found in patients with low malignant potential tumors (12.8 pg/mL) [47]. A further role of TF, either alone or in its complex, maybe to act as a ligand or tether for endothelial-anchored TFPI-1. The interaction of TF-TFPI-1 may act to halt the cancer cell in circulation and favor extravasation [48]. Although the consequence of a platelet-induced gain of TF in MICs (in this case 8% to 22%) is unknown, it may contribute to the clinical observation correlating platelet levels to an increase in cancer metastasis [21].

In summary, we report for the first time that platelets are present together with cancer cells in peritoneal ascites from ovarian cancer patients. We show that platelets can increase the levels of functional TF expression and MIC markers in ovarian cancer cells. Finally we demonstrate that platelets can act as a chemoattractant to cancer cells. This data complements the existing concept that platelets may be an interesting target for future cancer therapies.

## Conclusions

- Platelets potentiate a phenotypic change in ovarian cancer cells, inducing MIC (EMT/stem cell) markers
- Platelets act as chemoattractants to ovarian cancer cells
- Platelets promote ovarian cancer sphere formation.
- Platelets increase Tissue Factor, mRNA, protein and coagulating function.

## Additional files

Additional file 1: **Immunofluorescence negative control of CD42b.** Representative immunofluorescence from a smear of ascitic fluid obtained from ovarian cancer patients. (A) Absence of primary antibody CD42b (B) Vimentin staining in absence of primary antibody CD24b (C) Nuclear Hoechst staining. 
Additional file 2: **Activated platelets are present in ascites from ovarian cancer patients.** Flow cytometry analysis of CD61 of human ascites extracted from ovarian cancer patients. (A) Number of platelets detected (B) Representative plot of ascites content. Platelet events are surrounded by discontinuous line (C) Flow cytometry analysis of CD62P expressed in activated platelets of human ascites extracted from ovarian cancer patients. 
Additional file 3: **Platelets activation upon contact with the culture medium.** This graph demonstrates that the vast majority of the platelets added to each experiment become activated upon contact with the culture medium. This is not surprising, as it is known that platelets in the blood stream remain in an inactivated form due to the presence of inhibitors. We speculate that these inhibitors are diluted out once the platelets are placed in culture medium. However, it is also speculated that the activated platelets can possess growth factors (eg TGFbeta) that are anchored to the membrane and thus are not released. These require platelet-cancer cells interaction, where the cancer cells proteases can cleave the growth factors permitting a slow, maybe sustained, release. *p<0.05 with respect to control. N=3, Anova con Bonferroli post-test. There is no significant differences between the treatment conditions. 
Additional file 4: **Platelet presence does not alter the proliferation of the SKOV3 ovarian cancer cell line.** Cancer cell were treated with or without platelets (150,000/μL) for 12 hours before cell cycle evaluation by propidium iodide staining and flow cytometry analysis (M1, sub GO/GI (cell death). M2, GO/G1. M3, S/G2/M). Platelet presence produced no significant difference in cell cycle distribution. Each set of graphs (N) represents an independent experiment.
Additional file 5: **Ovarian cancer sphere isolation and characterization.** UCI101 ovarian, were cultured in 6 well low attachment plates in DMEM/F12 medium supplemented with FGF 50 ug/mL; EGF 0.2 mg/mL; insulin 5g/mL. Every 48 hours 1mL of supplemented medium with or without 1.5 10^5^ platelets uL^-1^ was added. After 7 day of culture, medium was passed through a strainer to select cancer spheres clusters of less than 100 μm. Spheres were recuperated and analyzed by immunocytochemistry in the presence and absence (negative control) of anti-CD44 antibody (A). Western blot analysis (B) and subsequent quantification (C) demonstrated an increase in CD44 and EMT markers and a decease in E-Cadherin in respect to the parental cell line (total population). Student t-test ,*p>0.05 respect to control, **p>0.01 respect to control (three independent experiments).

## Abbreviations

EMT: Epithelial-mesenchymal transition
CD44: Also referred as homing cell adhesion molecule (HCAM)
TF: Tissue factor
TGFβ: Transforming growth factor-beta
VEGF: vascular endothelial growth factor
PDGF: platelet–derived growth factor
Smad: Transcription factor that transduce extracellular signals from transforming growth factor beta ligands to the nucleus
NF-κB: Nuclear factor kappa-light-chain-enhancer of activated B cells (Transcription factor)
CD42b: Cluster of differentiation 42b
GPIb: Platelet glycoprotein Ib
TFPI: Tissue factor pathway inhibitor
YFP: Expressing yellow fluorescent protein
PGE1: Prostaglandin E1
RT-PCR: Reverse transcription polymerase chain reaction
