# Supplementary figures and images for: Platelets enhance tissue factor protein and metastasis initiating cell markers, and act as chemoattractants increasing the migration of ovarian cancer cells
Source: BMC Cancer. 2015 Apr 15;15:290. doi: 10.1186/s12885-015-1304-z (PMC4410584; doi:10.1186/s12885-015-1304-z)

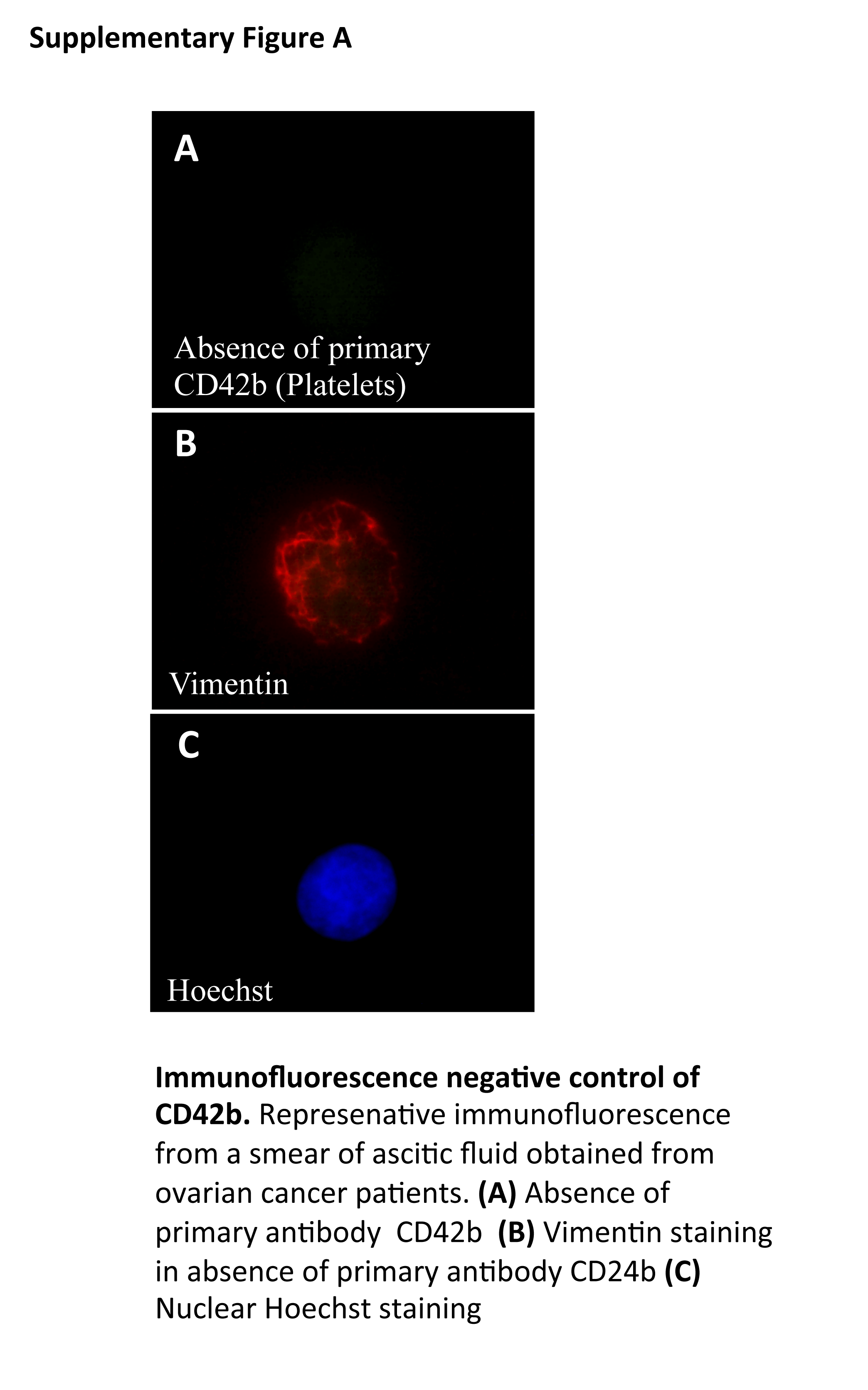

Supplement: Additional file 1: — Immunofluorescence negative control of CD42b. Representative immunofluorescence from a smear of ascitic fluid obtained from ovarian cancer patients. (A) Absence of primary antibody CD42b (B) Vimentin staining in absence of primary antibody CD24b (C) Nuclear Hoechst staining. [file 12885_2015_1304_MOESM1_ESM.png]

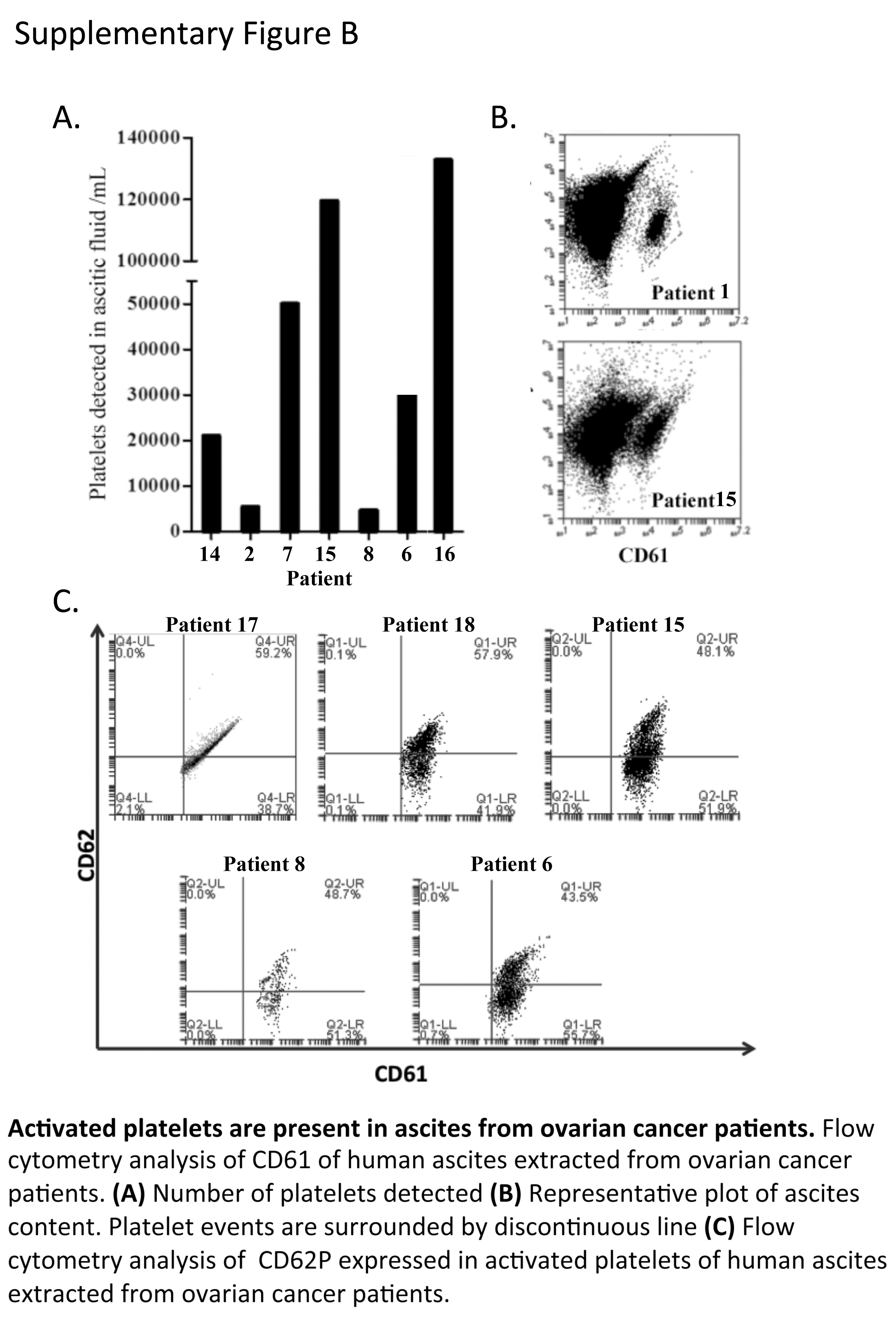

Supplement: Additional file 2: — Activated platelets are present in ascites from ovarian cancer patients. Flow cytometry analysis of CD61 of human ascites extracted from ovarian cancer patients. (A) Number of platelets detected (B) Representative plot of ascites content. Platelet events are surrounded by discontinuous line (C) Flow cytometry analysis of CD62P expressed in activated platelets of human ascites extracted from ovarian cancer patients. [file 12885_2015_1304_MOESM2_ESM.png]

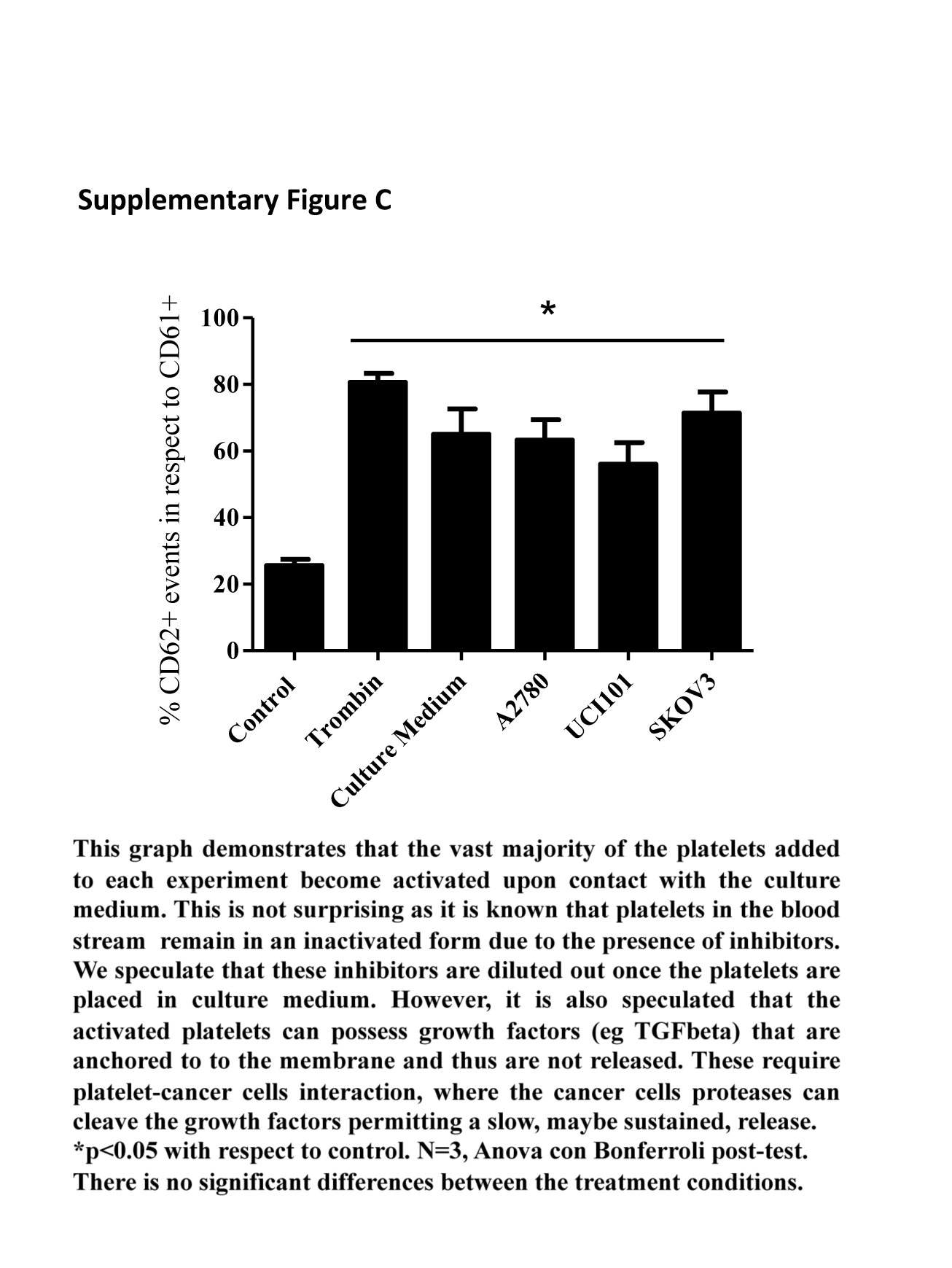

Supplement: Additional file 3: — Platelets activation upon contact with the culture medium. This graph demonstrates that the vast majority of the platelets added to each experiment become activated upon contact with the culture medium. This is not surprising, as it is known that platelets in the blood stream remain in an inactivated form due to the presence of inhibitors. We speculate that these inhibitors are diluted out once the platelets are placed in culture medium. However, it is also speculated that the activated platelets can possess growth factors (eg TGFbeta) that are anchored to the membrane and thus are not released. These require platelet-cancer cells interaction, where the cancer cells proteases can cleave the growth factors permitting a slow, maybe sustained, release. *p<0.05 with respect to control. N=3, Anova con Bonferroli post-test. There is no significant differences between the treatment conditions. [file 12885_2015_1304_MOESM3_ESM.png]

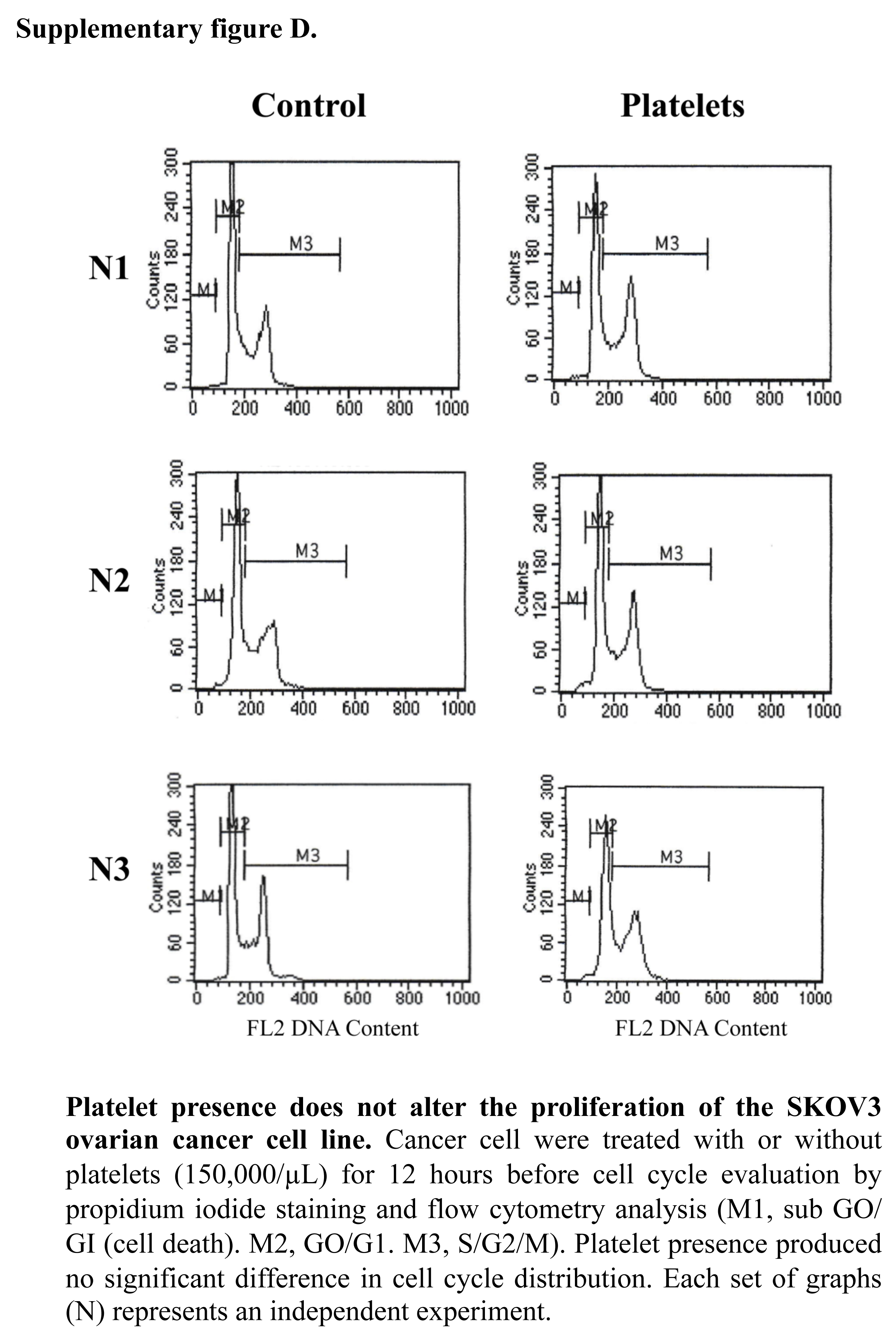

Supplement: Additional file 4: — Platelet presence does not alter the proliferation of the SKOV3 ovarian cancer cell line. Cancer cell were treated with or without platelets (150,000/μL) for 12 hours before cell cycle evaluation by propidium iodide staining and flow cytometry analysis (M1, sub GO/GI (cell death). M2, GO/G1. M3, S/G2/M). Platelet presence produced no significant difference in cell cycle distribution. Each set of graphs (N) represents an independent experiment. [file 12885_2015_1304_MOESM4_ESM.png]

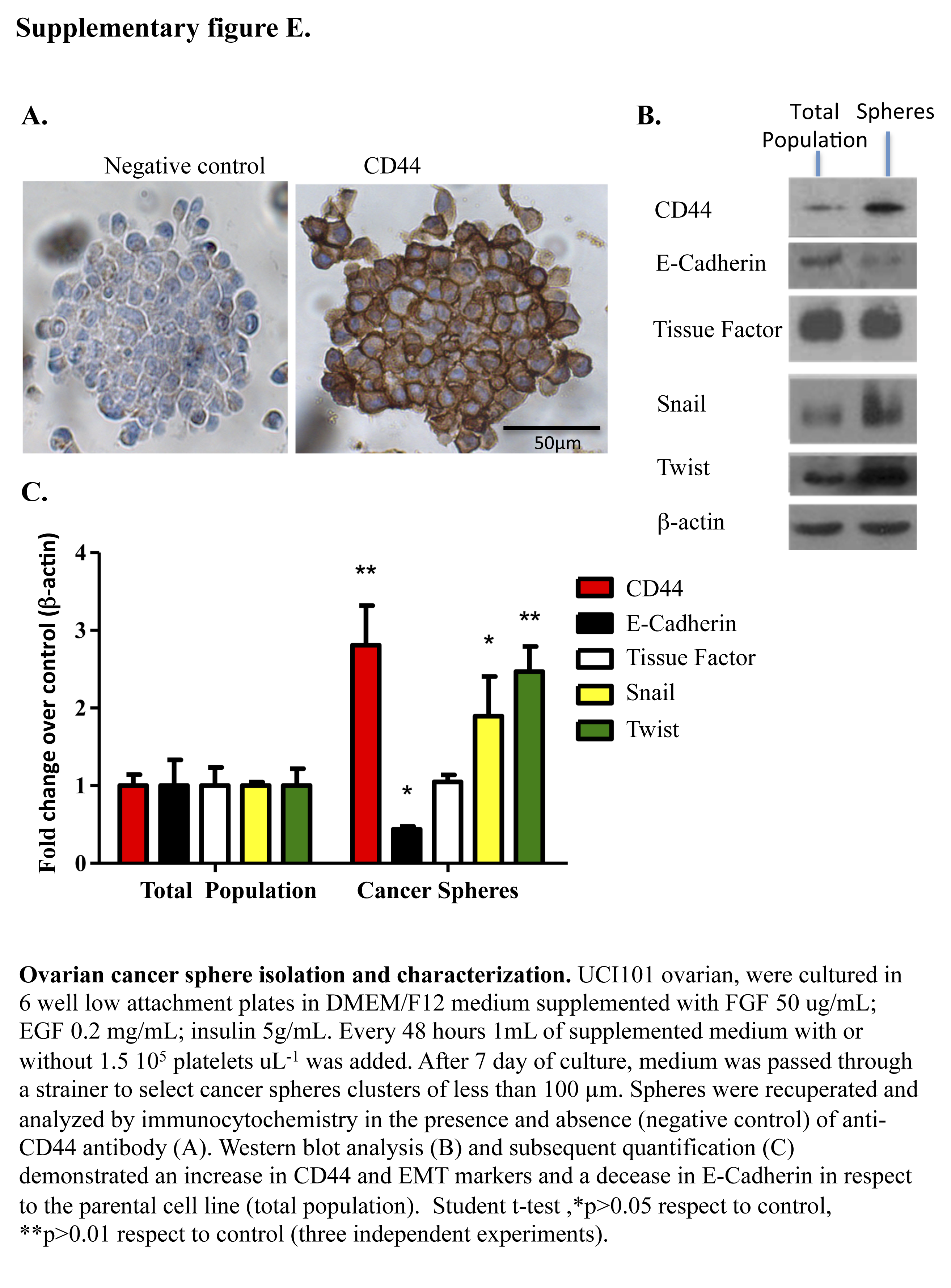

Supplement: Additional file 5: — Ovarian cancer sphere isolation and characterization. UCI101 ovarian, were cultured in 6 well low attachment plates in DMEM/F12 medium supplemented with FGF 50 ug/mL; EGF 0.2 mg/mL; insulin 5g/mL. Every 48 hours 1mL of supplemented medium with or without 1.5 105 platelets uL-1 was added. After 7 day of culture, medium was passed through a strainer to select cancer spheres clusters of less than 100 μm. Spheres were recuperated and analyzed by immunocytochemistry in the presence and absence (negative control) of anti-CD44 antibody (A). Western blot analysis (B) and subsequent quantification (C) demonstrated an increase in CD44 and EMT markers and a decease in E-Cadherin in respect to the parental cell line (total population). Student t-test ,*p>0.05 respect to control, **p>0.01 respect to control (three independent experiments). [file 12885_2015_1304_MOESM5_ESM.png]
